# Supplementary material for: Continuous high-fat high-sugar diet overrides the therapeutic potential of fecal microbiota transplantation from exercised and/or inulin-conditioned donors in obese mice
Source: PLoS One. 2026 May 12;21(5):e0349286. doi: 10.1371/journal.pone.0349286 (PMC13166953; doi:10.1371/journal.pone.0349286)
Supplement: S1 Appendix — (ZIP) [file pone.0349286.s001.zip › Underlying data for Fig 2.pdf]

"Donor" Body Weight (g)

		0wk	1wk	2wk	3wk	4wk	5wk	6wk	7wk	8wk	9wk	10wk	11wk
**Sed**	1	15.62	18.53	19.02	21.63	22.7	23.36	24.05	24.55	24.96	25.22	25.16	25.66
	2	16.8	19.47	21.02	22.79	23.84	25.16	26.05	25.92	26.62	27.07	26.65	27.41
	3	15.74	19.79	21.22	23.32	24.12	25.72	26.25	26.5	26.74	26.95	26.4	27.55
	4	16.03	19.65	20.58	23.15	23.91	25.49	25.81	26.02	25.81	26.14	26.48	27.4
	5	16.42	19.78	21.03	23.19	24.42	24.04	24.87	25.21	25.77	25.87	26.27	26.26
	6	14.36	18.39	18.82	21.88	22.84	25.48	26.62	26.44	26.59	26.98	27.7	27.56
	MEAN	15.83	19.27	20.28	22.66	23.64	24.88	25.61	25.77	26.08	26.37	26.44	26.97
	SD	0.84	0.64	1.08	0.73	0.70	0.95	0.96	0.76	0.69	0.75	0.81	0.81
	SE	0.34	0.26	0.44	0.30	0.29	0.39	0.39	0.31	0.28	0.31	0.33	0.33
**Ex**	1	15.23	16.82	17.01	18.76	19.55	20.58	21.45	21.65	22.3	22.79	23.18	23.31
	2	14.26	16.1	16.32	18.26	19.21	20.64	21.43	21.89	22.27	22.28	22.2	23.14
	3	14.86	18.23	17.34	19.95	20.57	21.51	22.16	22.34	22.65	22.91	23.31	23.67
	4	16.37	17.25	17.65	19.82	20.88	21.56	22.22	22.53	23.17	23.87	24.2	24.04
	5	15.07	18.26	18.03	21.4	22.24	23.26	23.63	24.64	24.97	25.5	26.28	26.54
	6	16.31	18.52	17.38	20.91	21.39	22.58	22.92	23.38	23.47	24.63	24.53	25.12
	MEAN	15.35	17.53	17.29	19.85	20.64	21.69	22.30	22.74	23.14	23.66	23.95	24.30
	SD	0.83	0.96	0.58	1.20	1.13	1.06	0.86	1.11	1.02	1.23	1.41	1.30
	SE	0.34	0.39	0.24	0.49	0.46	0.43	0.35	0.45	0.41	0.50	0.57	0.53
**Sed + Inu**	1	15.92	19.87	20.46	24.12	25.73	26.49	26.67	27.3	27.23	27.64	27.94	28.1
	2	15.98	19.1	19.95	23.53	24.69	26	26.83	27.24	27.49	28.08	28.58	29.58
	3	15.2	19.3	19.99	23.11	24.28	25.08	25.58	25.96	26.32	26.79	26.45	27.33
	4	16.12	19.87	21	24.79	26.31	27.73	28.55	28.47	28.62	28.7	29.19	30.01
	5	14.44	19.85	21.02	24.32	25.21	25.99	26.9	26.68	27.17	27.4	27.39	28.01
	6	14.95	19.51	18.9	23.57	24.8	26.32	27.15	27.32	27.8	28.42	28.6	28.89
	MEAN	15.44	19.58	20.22	23.91	25.17	26.27	26.95	27.16	27.44	27.84	28.03	28.65
	SD	0.68	0.33	0.80	0.61	0.74	0.87	0.96	0.83	0.76	0.70	0.99	1.02
	SE	0.28	0.14	0.33	0.25	0.30	0.35	0.39	0.34	0.31	0.29	0.40	0.42
**Ex + Inu**	1	15.16	18.65	17.88	20.84	20.98	22.51	22.62	23.59	23.96	24.86	24.88	25.74
	2	15.35	18.67	18.24	20.67	22.05	23.08	23.76	24.53	24.41	24.94	25.43	25.3
	3	16.51	18.09	17.8	20.99	21.88	23.26	23.49	23.77	23.97	24.36	24.63	24.46
	4	16.27	18.26	17.08	20.45	21.59	22.87	23.3	23.48	23.45	24.35	24.3	24.53
	5	16.18	18.68	18.14	20.67	21.46	22.7	23.04	23.54	23.73	23.5	23.46	23.6
	6	15.68	17.08	18.16	20.41	22.03	22.94	23.17	23.41	23.78	24.44	24.79	25.46
	MEAN	15.86	18.24	17.88	20.67	21.67	22.89	23.23	23.72	23.88	24.41	24.58	24.85
	SD	0.54	0.62	0.43	0.22	0.41	0.27	0.39	0.42	0.32	0.51	0.66	0.80
	SE	0.22	0.25	0.18	0.09	0.17	0.11	0.16	0.17	0.13	0.21	0.27	0.33

"Donor" Relative epididymal fat mass (%)

	**Sed**	**Ex**	**Sed + Inu**	**Ex + Inu**
1	1.87	1.20	1.64	1.09
2	2.12	1.01	2.06	1.31
3	2.12	1.66	1.82	1.25
4	2.39	1.41	1.48	1.43
5	2.42	1.78	1.87	1.37
6	2.68	1.88	1.47	1.01
Mean	2.27	1.49	1.72	1.24
SD	0.29	0.34	0.23	0.16
SE	0.12	0.14	0.10	0.07

"Donor" GTT

		0	15	30	60	120		Δ0-15	Δ15-30	Δ30-60	Δ60-120	AUC
**Sed**	1	143	405	425	245	182		1965	4080	5760	4230	16035
	2	163	349	346	234	180		1395	2767.5	3810	2640	10613
	3	119	213	270	195	173		705	1837.5	3405	3900	9848
	4	145	253	305	193	157		810	2010	3120	1800	7740
	5	148	422	306	212	173		2055	3240	3330	2670	11295
	6	151	280	245	222	178		967.5	1672.5	2475	2940	8055
	MEAN	144.83	320.33	316.17	216.83	173.83		1316.25	2601.25	3650.00	3030.00	10597.50
	SD	14.48	84.86	63.50	20.89	9.02		587.26	939.98	1122.64	894.38	3008.16
	SE	5.91	34.64	25.92	8.53	3.68		239.75	383.75	458.32	365.13	1228.08
**Ex**	1	130	360	333	194	132		1725	3247.5	4005	1980	10958
	2	150	307	334	193	131		1177.5	2557.5	3405	894.615	8035
	3	141	279	280	160	127		1035	2077.5	2370	328.225	5811
	4	141	234	267	163	124		697.5	1642.5	2220	372.35	4932
	5	132	283	239	218	140		1132.5	1935	2895	2820	8783
	6	160	395	282	204	162		1762.5	2677.5	2490	1380	8310
	MEAN	142.33	309.67	289.17	188.67	136.00		1255.00	2356.25	2897.50	1295.87	7804.62
	SD	11.25	58.65	37.62	22.91	13.84		414.41	583.54	691.00	974.51	2164.75
	SE	4.59	23.94	15.36	9.35	5.65		169.18	238.23	282.10	397.84	883.76
**Sed + Inu**	1	152	314	183	155	129		1215	1447.5	510	10.38	3183
	2	167	323	340	251	205		1170	2467.5	3855	3660	11153
	3	158	275	179	182	146		877.5	1035	675	360	2948
	4	129	273	242	205	167		1080	1927.5	2835	3420	9263
	5	158	251	225	189	144		697.5	1200	1470	640.615	4008
	6	130	310	265	230	164		1350	2362.5	3525	4020	11258
	MEAN	149.00	291.00	239.00	202.00	159.17		1065.00	1740.00	2145.00	2018.50	6968.50
	SD	15.85	28.61	59.70	34.56	26.45		238.83	604.33	1455.63	1862.60	4010.66
	SE	6.47	11.68	24.37	14.11	10.80		97.50	246.72	594.26	760.40	1637.35
**Ex + Inu**	1	157	269	304	150	133		840	1942.5	2105.04	0	4888
	2	130	279	258	178	138		1117.5	2077.5	2640	1680	7515
	3	132	236	202	169	161		780	1305	1605	1980	5670
	4	172	330	292	180	181		1185	2085	1920	510	5700
	5	130	293	139	156	140		1222.5	1290	525	1080	4118
	6	151	311	254	148	173		1200	1972.5	1501.225	0	4674
	MEAN	145.33	286.33	241.50	163.50	154.33		1057.50	1778.75	1716.04	875.00	5427.29
	SD	17.48	33.01	61.57	14.08	20.16		195.81	377.02	710.45	845.64	1188.83
	SE	7.14	13.48	25.13	5.75	8.23		79.94	153.92	290.04	345.23	485.34
