# Supplementary material for: Continuous high-fat high-sugar diet overrides the therapeutic potential of fecal microbiota transplantation from exercised and/or inulin-conditioned donors in obese mice
Source: PLoS One. 2026 May 12;21(5):e0349286. doi: 10.1371/journal.pone.0349286 (PMC13166953; doi:10.1371/journal.pone.0349286)
Supplement: S1 Appendix — (ZIP) [file pone.0349286.s001.zip › Underlying data for Fig 4.pdf]

ASVs

	Sham	Sed-R	Ex-R	Sed + Inu-R	Ex + Inu-R
1	98	201	205	206	188
2	113	202	207	190	198
3	89	202	204	223	204
4	105	184	237	242	222
5	96	191	227	177	214
6	69	180	198	184	222
7	85	182	208	210	186
8	81	200	184	217	198
MEAN	92.00	192.75	208.75	206.13	204.00
SD	13.99	9.63	16.47	21.72	14.14
SE	4.95	3.41	5.82	7.68	5.00

Chao 1 index

	Sham	Sed-R	Ex-R	Sed + Inu-R	Ex + Inu-R
1	98.00	213.65	207.04	214.71	189.95
2	115.09	206.15	220.03	199.03	200.11
3	90.00	204.91	216.73	237.02	209.31
4	109.07	188.95	240.64	248.28	224.86
5	99.00	196.11	235.05	180.32	217.88
6	72.00	183.00	201.06	188.52	229.00
7	86.50	183.00	214.19	221.51	195.00
8	85.20	203.00	187.00	226.82	203.00
MEAN	94.36	197.35	215.22	214.53	208.64
SD	13.86	11.44	17.44	23.70	14.16
SE	4.90	4.04	6.17	8.38	5.01

Shannon index

	Sham	Sed-R	Ex-R	Sed + Inu-R	Ex + Inu-R
1	3.40	4.35	4.90	4.31	4.12
2	3.70	4.15	4.11	3.54	4.04
3	3.31	4.90	4.21	3.77	3.84
4	3.61	3.95	5.33	4.25	4.09
5	3.17	4.25	4.34	3.37	3.90
6	3.02	4.80	4.29	3.50	4.31
7	3.75	4.64	4.10	3.91	4.04
8	3.71	4.76	3.87	3.81	4.21
MEAN	3.46	4.48	4.39	3.81	4.07
SD	0.28	0.35	0.48	0.34	0.15
SE	0.10	0.12	0.17	0.12	0.05

Simpson index

	Sham	Sed-R	Ex-R	Sed + Inu-R	Ex + Inu-R
1	0.73	0.84	0.91	0.84	0.86
2	0.80	0.84	0.81	0.77	0.84
3	0.76	0.90	0.86	0.78	0.81
4	0.77	0.80	0.93	0.83	0.82
5	0.70	0.84	0.86	0.77	0.83
6	0.70	0.92	0.88	0.75	0.83
7	0.81	0.90	0.85	0.82	0.85
8	0.85	0.91	0.81	0.79	0.85
MEAN	0.77	0.87	0.86	0.80	0.83
SD	0.06	0.05	0.04	0.03	0.02
SE	0.02	0.02	0.02	0.01	0.01

PCoA

"Donor"

PC1	Sed	Ex	Sed + Inu	Ex + Inu
1	0.01	0.00	-0.03	-0.13
2	0.00	-0.01	-0.02	-0.04
3	-0.01	0.03	-0.04	-0.08
4	-0.07	0.01	-0.08	-0.06
5	-0.02	0.03	-0.04	-0.06
6	-0.01	0.04	-0.07	-0.06
MEAN	-0.02	0.02	-0.05	-0.07
SD	0.03	0.02	0.02	0.03
SE	0.01	0.01	0.01	0.01

PC2	Sed	Ex	Sed + Inu	Ex + Inu
1	-0.15	-0.13	-0.20	-0.22
2	-0.14	-0.15	-0.17	-0.19
3	-0.15	-0.06	-0.20	-0.22
4	-0.22	-0.13	-0.21	-0.20
5	-0.12	-0.12	-0.21	-0.22
6	-0.19	-0.14	-0.21	-0.19
MEAN	-0.16	-0.12	-0.20	-0.21
SD	0.04	0.03	0.02	0.02
SE	0.01	0.01	0.01	0.01

PC3	Sed	Ex	Sed + Inu	Ex + Inu
1	-0.05	-0.07	-0.05	0.00
2	-0.04	-0.04	-0.03	-0.06
3	-0.04	-0.04	-0.04	-0.03
4	-0.02	-0.06	-0.09	-0.04
5	-0.03	-0.05	-0.05	-0.04
6	-0.05	-0.05	-0.07	-0.04
MEAN	-0.04	-0.05	-0.05	-0.03
SD	0.01	0.01	0.02	0.02
SE	0.01	0.00	0.01	0.01

"Recipient" pre

PC1	Sham	Sed-R	Ex-R	Sed + Inu-R	Ex + Inu-R
1	-0.26	0.04	0.06	0.05	0.04
2	-0.25	0.07	0.03	0.03	0.01
3	-0.34	0.04	0.04	0.05	-0.01
4	-0.29	0.05	0.04	0.05	0.02
5	-0.31	0.06	0.08	0.00	0.06
6	-0.42	0.05	0.07	0.05	0.06
7	-0.34	0.05	0.05	0.02	0.02
8	-0.37	0.06	0.03	0.04	0.05
MEAN	-0.32	0.05	0.05	0.04	0.03
SD	0.06	0.01	0.02	0.02	0.02
SE	0.02	0.00	0.01	0.01	0.01

PC2	Sham	Sed-R	Ex-R	Sed + Inu-R	Ex + Inu-R
1	0.04	0.01	0.00	-0.02	-0.05
2	0.02	0.00	0.00	-0.04	-0.05
3	0.02	0.01	0.00	-0.03	-0.08
4	0.04	-0.02	0.03	-0.01	-0.01
5	0.01	-0.01	-0.01	-0.09	-0.05
6	0.02	0.00	-0.03	-0.01	-0.05
7	-0.03	-0.07	-0.04	-0.07	-0.08
8	-0.03	0.02	-0.03	-0.03	-0.06
MEAN	0.01	-0.01	-0.01	-0.04	-0.05
SD	0.03	0.03	0.02	0.03	0.02
SE	0.01	0.01	0.01	0.01	0.01

PC3	Sham	Sed-R	Ex-R	Sed + Inu-R	Ex + Inu-R
1	0.30	0.01	-0.01	-0.01	-0.02
2	0.27	-0.01	-0.02	-0.03	-0.03
3	0.28	0.05	0.01	-0.04	-0.01
4	0.28	0.00	0.01	-0.05	-0.03
5	0.28	-0.02	-0.02	-0.03	-0.06
6	0.33	0.02	-0.02	-0.02	-0.03
7	0.25	0.01	-0.03	-0.07	-0.03
8	0.26	0.03	-0.02	-0.03	-0.03
MEAN	0.28	0.01	-0.01	-0.04	-0.03
SD	0.02	0.02	0.01	0.02	0.01
SE	0.01	0.01	0.01	0.01	0.00

"Recipient" post

PC1	Sham	Sed-R	Ex-R	Sed + Inu-R	Ex + Inu-R
1	0.12	0.10	0.09	0.09	0.06
2	0.13	0.10	0.07	0.11	0.09
3	0.11	0.12	0.13	0.10	0.11
4	0.11	0.10	0.10	0.06	0.08
5	0.11	0.12	0.10	0.10	0.09
6	0.11	0.10	0.05	0.09	0.11
7	0.10	0.08	0.11	0.09	0.09
8	0.12	0.12	0.11	0.09	0.08
MEAN	0.11	0.11	0.09	0.09	0.09
SD	0.01	0.01	0.03	0.01	0.02
SE	0.00	0.00	0.01	0.01	0.01

PC2	Sham	Sed-R	Ex-R	Sed + Inu-R	Ex + Inu-R
1	0.06	0.10	0.10	0.07	0.12
2	0.13	0.11	0.12	0.08	0.12
3	0.09	0.08	0.10	0.11	0.08
4	0.08	0.11	0.09	0.10	0.15
5	0.04	0.12	0.11	0.13	0.09
6	0.12	0.11	0.06	0.11	0.09
7	0.11	0.09	0.08	0.12	0.10
8	0.10	0.09	0.07	0.10	0.11
MEAN	0.09	0.10	0.09	0.10	0.11
SD	0.03	0.01	0.02	0.02	0.02
SE	0.01	0.00	0.01	0.01	0.01

PC3	Sham	Sed-R	Ex-R	Sed + Inu-R	Ex + Inu-R
1	-0.03	0.04	-0.01	0.03	-0.01
2	0.00	0.03	0.06	0.03	0.03
3	-0.01	0.02	-0.03	0.01	0.00
4	0.04	0.04	0.05	0.03	0.06
5	-0.01	0.00	-0.01	0.02	0.02
6	0.02	0.02	0.00	0.05	0.01
7	0.04	0.01	0.00	0.02	0.00
8	0.00	-0.01	0.01	0.03	0.02
MEAN	0.01	0.02	0.01	0.03	0.02
SD	0.02	0.02	0.03	0.01	0.02
SE	0.01	0.01	0.01	0.00	0.01
