# Supplementary material for: Continuous high-fat high-sugar diet overrides the therapeutic potential of fecal microbiota transplantation from exercised and/or inulin-conditioned donors in obese mice
Source: PLoS One. 2026 May 12;21(5):e0349286. doi: 10.1371/journal.pone.0349286 (PMC13166953; doi:10.1371/journal.pone.0349286)
Supplement: S1 Appendix — (ZIP) [file pone.0349286.s001.zip › Underlying data for Fig 6.pdf]

"Recipient" Body weight (g)

		0 wk	1 wk	2 wk	3 wk	4 wk	5 wk	6 wk	7 wk	8 wk	9 wk	10 wk	11 wk	12 wk	Abx	FMT 1 wk	FMT 2 wk	FMT 3 wk	FMT 4 wk
**Sham**	1	15.2	21.57	23.2	25.62	28.02	30.86	34.03	35.43	37.69	39.47	41.45	42.27	42.34	42.17	40.74	39.81	40.04	39.66
	2	14.71	20.31	22.1	24.54	26.4	28.72	31.6	33.58	36.98	38.51	41.99	40.25	41.78	42.22	40.35	39.24	39.56	38.78
	3	14.78	21.06	22.92	25.14	27.72	29.54	32.76	33.91	37.4	40.17	42.76	42.6	43.92	43.9	42.51	41.57	41.1	40.91
	4	13.45	19.35	20.94	22.47	26.37	25.71	27.44	28.12	30.33	31.12	33.43	33.61	33.98	33.79	32.92	33.15	33.03	33.32
	5	13.48	19.29	20.82	22.32	23.96	24.81	27.04	28.8	30	31.73	33.63	34.24	34.62	35	34.04	33.31	32.2	31.31
	6	14.59	21.09	22.34	23.41	25.36	26.84	29.87	31.76	33.83	36.8	39.46	39.89	40.01	40.77	39.15	38.54	38.42	37.66
	7	14.55	20.24	21.36	23.55	25.06	27.62	30.21	32.03	34.16	35.71	37.63	37.08	38.4	38.38	38.86	37.85	37.5	36.82
	8	13.56	19.81	21	22.71	24.64	26.47	28.52	30.56	34.44	36.9	39.18	40.37	40.15	41.93	42.07	40.9	42.39	42.93
	9	14.41	20.02	20.88	22.59	24.71	27.25	30.11	32.06	34.19	36.98	39.04	39.51	41.74	41.41	40.76	40.55	40.1	40.03
	10	13.75	19.69	21.25	22.99	24.42	26.91	28.78	30.47	33.29	35.48	37.52	37.09	37.92	38.18	37.32	36.82	37.06	36.84
	11	15.98	20.57	22.51	23.48	25.18	27.7	29.96	31.42	33.71	35.85	37.27	37.6	39.08	38.41	36.31	35.93	35.72	36.13
	12	13.13	20.81	23.55	25.4	27.34	29.28	31.44	33.67	35.74	38.37	39.73	38.79	40.51	41.09	40.51	40.09	40.53	40.54
	MEAN	14.30	20.32	21.91	23.69	25.77	27.64	30.15	31.82	34.31	36.42	38.59	38.61	39.54	39.77	38.80	38.15	38.14	37.91
	SD	0.84	0.72	0.99	1.19	1.37	1.71	2.07	2.14	2.45	2.76	2.94	2.82	2.98	3.07	3.06	2.83	3.18	3.30
	SE	0.24	0.21	0.28	0.34	0.40	0.49	0.60	0.62	0.71	0.80	0.85	0.81	0.86	0.89	0.88	0.82	0.92	0.95
**Sed-R**	1	14.6	19.62	21.31	23.72	25.93	28.87	31.3	33.94	36.12	38.64	41.27	41.43	41.54	40.95	40.77	40.72	40.81	40.83
	2	13.9	19.22	19.97	21.9	23.84	25.04	27.4	29.23	31.25	32.98	35.04	35.23	35.62	36.24	35.33	34.44	34.13	33.91
	3	14.28	20.26	22.81	25.12	27.67	30.16	34.13	36.84	40.16	42.92	45.39	45.85	46.81	47.2	45.46	44.9	44.34	44.34
	4	13.63	19.32	20.37	21.96	23.12	24.78	26.58	28.63	30.13	31.76	33.84	35.6	37.36	38.91	38.3	37.75	37.44	37.29
	5	14.08	20.27	21.58	23.13	25.15	27.15	29.56	32.02	34.24	35.97	38.18	38.82	40.51	40.99	39.66	39.57	39.73	39.55
	6	13.38	20.76	23.02	24.34	27	29.43	31.72	34.62	36.7	38.51	40.09	40.32	41.28	41.33	40.56	41.09	41.5	42.3
	7	13.29	18.63	20.28	22.12	23.4	26.13	27.74	30.26	31.84	33.16	35.16	34.94	36.1	36.5	34.78	34.85	34.6	33.7
	8	14.96	20.17	21.84	23.49	26.25	28.24	31.07	33.51	35.7	37.18	39.2	38.88	40.75	41.58	39.27	39.65	39.47	39
	9	14.69	21.9	22.87	24.87	27.35	29.89	32.58	34.21	37.41	39.12	41.42	41.67	42.9	43.05	41.56	41	40.45	40.16
	10	14.38	21.04	22.33	24.42	27	29.82	32.33	34.11	36.63	39.42	42.55	43.12	44.5	45.38	43.88	43.4	43.15	43.39
	11	14.62	21.12	22	23.57	25.63	27.52	30.02	32.06	34.83	36.09	39.2	39.48	40.81	42.35	40.46	40.81	40.63	41.12
	12	13.05	20.34	22.28	24.7	27.21	29.34	32.71	36.17	38.25	40.03	43.28	42.78	43.44	44.27	42.65	43.01	42.4	41.85
	MEAN	14.07	20.22	21.72	23.61	25.80	28.03	30.60	32.97	35.27	37.15	39.55	39.84	40.97	41.56	40.22	40.10	39.89	39.79
	SD	0.62	0.92	1.05	1.14	1.60	1.90	2.37	2.59	2.98	3.30	3.54	3.40	3.34	3.28	3.13	3.17	3.14	3.38
	SE	0.18	0.27	0.30	0.33	0.46	0.55	0.68	0.75	0.86	0.95	1.02	0.98	0.96	0.95	0.90	0.92	0.91	0.98
**Ex-R**	1	13.61	21.85	23.44	25.03	28.36	31.82	35.51	37.69	40.17	43.29	45.38	47.08	48.24	48.61	47.87	47.97	47.78	47.95
	2	13.96	20.25	22.49	24.27	25.62	27.57	29.2	30.63	32.25	33.34	35.39	35.19	35.3	35.83	33.99	33.11	32.34	31.96
	3	14.74	21.62	23.17	25.04	26.72	29.69	32.6	34.15	35.95	37.74	39.7	41.04	42.59	42.77	41.04	42.57	42.21	41.39
	4	13.69	20.81	22.04	23.54	26.13	28.86	31.57	34.31	36.49	38.1	40.18	41.42	42.7	43.16	41.99	42.48	42.87	42.8
	5	12.91	19.12	20.54	22.38	24.02	25.66	27.82	29.72	31.75	33.65	35.76	35.83	36.82	35.06	33.64	34.33	34.92	34.36
	6	13.14	18.88	20.18	21.39	22.62	24.3	26.6	28.12	30.45	31.7	33.57	34.12	36.33	36.32	34.63	33.82	34.3	32.78
	7	14.04	20.1	21.94	24.1	27.04	30.19	33.21	35.63	37.36	39.82	41.83	41.14	44.22	44.12	43.8	43.57	43.23	42.43
	8	14.24	21.3	25.14	26.96	29.87	33.27	36.3	39.2	41.03	43.81	46.25	47.34	47.15	48.19	47.55	47.25	47.01	46.87
	9	14.69	21.99	23.34	25.3	28.36	31.35	33.73	36.27	38.22	40.83	42.73	43.28	45.61	46.08	43.28	43.32	43.25	42.75
	10	14.35	19.4	21.69	23.02	25.11	28.19	31.16	33.32	35.28	37.76	39.56	40.4	42.58	43.37	41.94	42.46	42	41.66
	11	15.01	21.01	23.49	25.42	28.74	32	34.08	36.65	38.47	40.7	42.57	43.51	44.5	44.84	43.29	43.15	42.79	42.4
	12	14.44	20.11	21.97	23.34	25.64	29.08	30.72	32.5	34.58	36.34	37.76	38.25	40.05	40.9	38.24	39.11	38.13	38.08
	MEAN	14.07	20.54	22.45	24.15	26.52	29.33	31.88	34.02	36.00	38.09	40.06	40.72	42.17	42.44	40.94	41.10	40.90	40.45
	SD	0.64	1.06	1.37	1.52	2.10	2.65	2.97	3.34	3.32	3.86	3.95	4.30	4.24	4.60	4.88	4.98	4.92	5.15
	SE	0.19	0.31	0.40	0.44	0.61	0.77	0.86	0.96	0.96	1.11	1.14	1.24	1.23	1.33	1.41	1.44	1.42	1.49
**Sed + Inu-R**	1	14.24	20.93	23.79	25.68	27.66	29.96	33.83	35.7	38.68	41.29	44.13	44.7	46.61	46.84	46.31	46.32	46.67	46.22
	2	15.52	20.78	22.16	23.84	26.66	30.14	34.86	36.98	40.73	43.41	46.35	43.04	45.76	47.15	47.08	48.11	48.25	47.8
	3	14.53	20.42	21.18	22.74	23.41	25.23	26.89	28.54	29.99	31.96	35.51	35.85	36.09	35.8	34.04	33.64	32.56	32.16
	4	14.13	20.96	21.9	23.89	26.11	28.54	31.51	32.92	34.63	37.1	39.16	40.11	41.75	42.57	41.75	41.81	41.99	41.85
	5	12.96	19.73	21.58	23.51	25.48	28.07	29.96	32.19	35.22	36.97	39.65	39.7	41.21	41.4	37.99	39.15	39.16	39.42
	6	13.21	20.94	22.95	24.92	27.81	30.87	33.06	35.74	38.91	41.62	44.47	45.25	46.1	47.52	45.48	46.11	45.47	45.09
	7	13.02	20.89	21.93	23.72	26.14	29.18	31.13	33.49	36.34	38.72	39.77	40.91	42.65	39.05	37.38	36.99	35.7	34.78
	8	14.47	19.57	20.9	22.54	24.49	27.3	29.92	31.54	34.46	36.65	38.58	38.68	40.24	42.59	41.88	42.89	42.58	43.59
	9	12.73	17.24	19.94	20.68	22.42	24.94	26.93	28.25	29.91	31.65	34.13	34.88	36.12	35.54	33.68	32.89	31.88	31.05
	10	13.61	20.57	21.9	23.71	26.23	29.54	32.2	34.69	36.55	39.15	42.72	43.02	44.71	44.87	43.93	43.51	42.92	43.41
	11	13.41	19.5	20.83	22.7	24.09	26.31	28.21	29.76	31.07	33.27	36.19	36.65	37.24	36.88	35.28	34.94	34.56	34.12
	12	15.14	20.31	21.33	23.09	24.75	27.89	29.84	31.35	33.84	35.79	38.75	39.93	40.44	40.61	39.3	38.58	38.76	37.93
	MEAN	13.91	20.15	21.70	23.42	25.44	28.16	30.70	32.59	35.03	37.30	39.95	40.23	41.58	41.74	40.34	40.41	40.04	39.79
	SD	0.90	1.07	1.01	1.25	1.65	1.93	2.57	2.86	3.49	3.78	3.79	3.37	3.77	4.33	4.76	5.16	5.50	5.73
	SE	0.26	0.31	0.29	0.36	0.48	0.56	0.74	0.83	1.01	1.09	1.10	0.97	1.09	1.25	1.38	1.49	1.59	1.65
**Ex + Inu-R**	1	14.09	19.97	21.33	22.78	24.92	27.26	29.01	31.11	33.05	34.93	38.86	38.79	41.41	41.42	42.1	42.81	43.01	43.28
	2	14.7	21.04	23.13	25.04	27.04	30	31.65	33.83	36.26	38.84	42.17	42.57	44.67	44.36	42.45	41.5	41.87	41.31
	3	13.84	18.19	19.97	21.52	23.84	25.65	28.2	30.49	33.4	35.73	38.61	39.72	41.35	41.59	40.01	41.1	41.53	41.07
	4	13.1	19.76	21.95	23.38	25.05	27.64	29.56	31.37	32.86	34.19	36.53	36.96	39.28	39.64	37.94	38.81	38.84	37.98
	5	12.71	20.13	21.87	23.84	26.08	28.74	31.32	33.1	35.96	38.08	41.05	42.18	43.36	43.2	42.26	43.16	43.39	42.17
	6	14.3	20.88	23.09	25.07	27.44	29.87	31.87	34.31	36.93	38.72	41.11	42.22	44	43.25	42.69	42.27	41.92	41.5
	7	13.79	20.28	21.64	23.37	24.62	26.56	28.78	30.12	32.27	33.34	35.21	35.48	36.39	37.1	35.96	36.17	36.07	36.51
	8	14.45	20.64	22.12	23.27	25.54	28.28	29.87	31.27	33.5	35.34	36.71	37.02	37.22	37.87	36.69	36.67	36.47	36.23
	9	13.78	20.13	21.68	23.16	24.78	27.19	28.96	30.95	32.28	34.19	35.17	35.14	35.12	35.19	34	33.23	33.23	33.19
	10	14.72	20.28	21.76	23.38	25.37	28.33	31.07	33.54	36.16	38.4	39.56	40.5	40.99	40.79	39.99	40.35	39.15	38.24
	11	13.55	20.17	21.84	23.84	25.42	28.12	30.78	32.54	34.07	36.04	36.87	37.68	39.35	38.48	35.69	35.31	34.95	35.5
	12	14.91	21.61	22.9	24.91	27	30.18	32.46	34.79	37.46	39.4	40.78	40.25	42.95	43.64	42.57	43.08	42.46	42.49
	MEAN	14.00	20.26	21.94	23.63	25.59	28.15	30.29	32.29	34.52	36.43	38.55	39.04	40.51	40.54	39.36	39.54	39.41	39.12
	SD	0.67	0.83	0.86	1.02	1.10	1.40	1.41	1.60	1.90	2.13	2.43	2.61	3.09	2.91	3.17	3.42	3.48	3.27
	SE	0.19	0.24	0.25	0.30	0.32	0.41	0.41	0.46	0.55	0.62	0.70	0.75	0.89	0.84	0.92	0.99	1.00	0.94

"Recipient" Relative epididymal fat mass (%)

	Sham	Sed-R	Ex-R	Sed + Inu-R	Ex + Inu-R
1	4.51	6.63	4.56	4.14	5.21
2	5.02	6.02	4.26	4.28	4.91
3	4.35	3.53	5.46	5.05	5.06
4	5.83	5.46	4.42	5.21	6.32
5	5.04	6.69	4.95	5.49	4.96
6	6.02	4.65	6.70	4.73	4.81
7	5.64	5.49	4.67	5.06	5.48
8	4.40	5.84	3.42	6.68	5.74
9	5.97	4.72	3.88	4.68	4.99
10	5.59	3.96	4.67	4.45	5.26
11	6.21	5.57	4.45	5.11	5.52
12	6.14	4.48	6.60	5.84	5.68
Mean	5.39	5.25	4.84	5.06	5.33
SD	0.70	1.00	0.99	0.71	0.44
SE	0.20	0.29	0.28	0.20	0.13

"Recipient" GTT

Pre		0	15	30	60	120		Δ0-15	Δ15-30	Δ30-60	Δ60-120	AUC
**Sham**	1	229	422	812	549	401		1447.5	5820	13545	14760	35573
	2	245	474	696	579	335		1717.5	5100	11775	12720	31313
	3	231	449	545	744	470		1635	3990	12405	22560	40590
	4	166	394	573	518	285		1710	4762.5	11385	14130	31988
	5	177	307	698	403	243		975	4882.5	11205	8760	25823
	6	182	346	592	542	470		1230	4305	11550	19440	36525
	7	211	395	708	569	356		1380	5107.5	12825	15090	34403
	8	245	416	502	494	354		1282.5	3210	7590	10740	22823
	9	225.5	507	736	505	337		2111.25	5940	11850	11730	31631
	10	171.5	304	531	393	326		993.75	3690	8715	11280	24679
	11	262	465	926	578	375		1522.5	6502.5	14700	12870	35595
	12	202.5	450	560	556	330		1856.25	4537.5	10665	14430	31489
	MEAN	212.29	410.75	656.58	535.83	356.83		1488.44	4820.63	11517.50	14042.50	31869.06
	SD	32.31	64.84	128.47	90.33	66.44		340.90	960.89	1931.45	3794.31	5242.97
	SE	9.33	18.72	37.09	26.08	19.18		98.41	277.38	557.56	1095.32	1513.51
**Sed-R**	1	165	418	583	475	330		1897.5	5032.5	10920	14250	32100
	2	207	392	553	580	271		1387.5	3982.5	10785	13110	29265
	3	213	430	588	806	405		1627.5	4440	14520	23550	44138
	4	234	455	868	594	413		1657.5	6412.5	14910	16170	39150
	5	187	385	744	501	245		1485	5662.5	13065	11160	31373
	6	216	426	650	535	365		1575	4830	11295	14040	31740
	7	240	427	842	838	470		1402.5	5917.5	18000	24840	50160
	8	179	422	760	541	288		1822.5	6180	14145	14130	36278
	9	201	466	530	666	329		1987.5	4455	11910	17790	36143
	10	229	473	535	578	371		1830	4125	9825	14730	30510
	11	194	380	599	518	225		1395	4432.5	10935	10650	27413
	12	228	443	592	670	413		1612.5	4342.5	12090	18810	36855
	MEAN	207.75	426.42	653.67	608.50	343.75		1640.00	4984.38	12700.00	16102.50	35426.88
	SD	23.37	30.13	119.28	116.09	75.76		205.85	845.37	2329.54	4457.53	6588.91
	SE	6.75	8.70	34.43	33.51	21.87		59.42	244.04	672.48	1286.78	1902.05
**Ex-R**	1	221	413	455.5	548	437		1440	3198.75	8422.5	16290	29351
	2	166.5	404	594	510	308		1781.25	4987.5	11565	14550	32884
	3	197	454	762	500	304		1927.5	6165	13020	12300	33413
	4	197	477	462.5	525	283		2100	4091.25	8902.5	12420	27514
	5	187	462	808	784	292		2062.5	6720	18270	21060	48113
	6	225	452	764	576	352		1702.5	5745	13350	14340	35138
	7	204	442	571	551	350		1785	4537.5	10710	14790	31823
	8	227	408	320	220.5	193		1357.5	2055	1297.5	0	4710
	9	172	341	327	229	208		1267.5	2430	3180	2790	9668
	10	219	480	569	567	381		1957.5	4582.5	10470	15300	32310
	11	212	372	521	409	249		1200	3517.5	7590	7020	19328
	12	194	268.5	303	201	195		558.75	1376.25	1740	240	3915
	MEAN	201.79	414.46	538.08	468.38	296.00		1595.00	4117.19	9043.13	10925.00	25680.31
	SD	20.02	62.42	175.66	174.20	76.46		448.78	1667.45	5053.33	6805.49	13528.04
	SE	5.78	18.02	50.71	50.29	22.07		129.55	481.35	1458.77	1964.57	3905.21
**Sed + Inu-R**	1	196	408	590	599	532		1590	4545	11955	22170	40260
	2	218	479	532	790	387		1957.5	4312.5	13290	22230	41790
	3	172.5	441	481	462	235		2013.75	4327.5	8970	10560	25871
	4	238	342	297.5	202	243		780	1226.25	352.5	0	2359
	5	248	487	868	520	339		1792.5	6442.5	13380	10890	32505
	6	192	308	282.5	198	254		870	1548.75	1447.5	2040	5906
	7	219	433	547	533	277		1605	4065	9630	11160	26460
	8	227	422	816	550	365		1462.5	5880	13680	13830	34853
	9	166	451	818	564	291		2137.5	7027.5	15750	15690	40605
	10	180	523	570	537	364		2572.5	5497.5	11205	16230	35505
	11	177.5	369	766	452	297		1436.25	5850	12945	11820	32051
	12	252	525	856	584	401		2047.5	6577.5	14040	14430	37095
	MEAN	207.17	432.33	618.67	499.25	332.08		1688.75	4775.00	10553.75	12587.50	29605.00
	SD	30.37	67.91	206.67	163.53	84.90		515.89	1858.04	4888.39	6687.49	12946.27
	SE	8.77	19.60	59.66	47.21	24.51		148.93	536.37	1411.16	1930.51	3737.27
**Ex + Inu-R**	1	203	429	728	506	298		1695	5632.5	12420	11940	31688
	2	196.5	440	584	547	330		1826.25	4732.5	11070	14520	32149
	3	223	477	718	508	341		1905	5617.5	11700	12090	31313
	4	198	351	561	575	354		1147.5	3870	11100	15990	32108
	5	212	499	506	413	294		2152.5	4357.5	7425	8490	22425
	6	213	495	836	804	348		2115	6787.5	18210	21780	48893
	7	151	332	495	504	289		1357.5	3937.5	10455	14730	30480
	8	191	441	770	495	346		1875	6217.5	13245	13770	35108
	9	178.5	388	581	563	268		1571.25	4590	11805	14220	32186
	10	191	362	583	588	245		1282.5	4222.5	11835	13530	30870
	11	185	409	570	478	269		1680	4567.5	10170	11310	27728
	12	194	408	588	545	359		1605	4560	11175	15480	32820
	MEAN	194.67	419.25	626.67	543.83	311.75		1684.38	4924.38	11717.50	13987.50	32313.75
	SD	18.62	54.93	108.59	94.93	39.21		314.14	926.07	2495.64	3209.23	6095.19
	SE	5.37	15.86	31.35	27.40	11.32		90.68	267.33	720.43	926.42	1759.53

Post		0	15	30	60	120		Δ0-15	Δ15-30	Δ30-60	Δ60-120	AUC
**Sham**	1	244	422	668	545	281		1335	4515	10875	10140	26865
	2	239	476	644	497	257		1777.5	4815	9945	8280	24818
	3	235	436	860	752	320		1507.5	6195	17130	18060	42893
	4	149	438	676	688	266		2167.5	6120	15990	19680	43958
	5	159	404	694	509	300		1837.5	5850	13275	14730	35693
	6	137	299	580	497	332		1215	4537.5	12045	16650	34448
	7	177	366	704	511	293		1417.5	5370	12915	13500	33203
	8	212	451	664	503	331		1792.5	5182.5	11145	12300	30420
	9	183	428	608	459	270		1837.5	5025	10515	10890	28268
	10	169	315	718	504	329		1095	5212.5	13260	14850	34418
	11	175	375	642	520	310		1500	5002.5	12180	14400	33083
	12	174	406	644	452	308		1740	5265	11220	12360	30585
	MEAN	187.75	401.33	675.17	536.42	299.75		1601.88	5257.50	12541.25	13820.00	33220.63
	SD	36.13	53.62	70.05	90.25	26.46		308.71	554.23	2170.88	3301.72	5785.74
	SE	10.43	15.48	20.22	26.05	7.64		89.12	159.99	626.68	953.12	1670.20
**Sed-R**	1	198	437	724	498	209		1792.5	5737.5	12390	9330	29250
	2	137	458	690	508	282		2407.5	6555	13860	15480	38303
	3	222	442	648	466	263		1650	4845	10050	8550	25095
	4	196	377	638	575	412		1357.5	4672.5	12315	17850	36195
	5	137	300	714	534	401		1222.5	5550	14610	19830	41213
	6	181	421	576	490	368		1800	4762.5	10560	14880	32003
	7	158	428	692	545	308		2025	6030	13815	16110	37980
	8	138	323	644	499	279		1387.5	5182.5	13005	15060	34635
	9	174	367	640	532	347		1447.5	4942.5	12360	15930	34680
	10	186	374	628	503	269		1410	4725	11385	12000	29520
	11	198	410	718	480	239		1590	5490	12030	9690	28800
	12	190	460	612	455	247		2025	5190	10305	9660	27180
	MEAN	176.25	399.75	660.33	507.08	302.00		1676.25	5306.88	12223.75	13697.50	32904.38
	SD	27.99	51.91	46.68	34.36	65.71		347.59	585.54	1461.14	3728.29	5019.28
	SE	8.08	14.99	13.47	9.92	18.97		100.34	169.03	421.80	1076.27	1448.94
**Ex-R**	1	217	486	618	536	304		2017.5	5025	10800	12180	30023
	2	186	466	656	510	238		2100	5625	11910	11280	30915
	3	153	440	684	531	293		2152.5	6135	13635	15540	37463
	4	236	534	786	800	352		2235	6360	16710	20400	45705
	5	199	466	770	571	380		2002.5	6285	14145	16590	39023
	6	139	388	692	537	294		1867.5	6015	14265	16590	38738
	7	226	383	806	748	229		1177.5	5527.5	16530	15750	38985
	8	219	445	658	374	256		1695	4987.5	8910	5760	21353
	9	162	361	652	443	222		1492.5	5167.5	11565	10230	28455
	10	172	434	638	513	361		1965	5460	12105	15900	35430
	11	198	503	720	502	244		2287.5	6202.5	12390	10500	31380
	12	155	452	716	554	266		2227.5	6435	14400	15300	38363
	MEAN	188.50	446.50	699.67	551.58	286.58		1935.00	5768.75	13113.75	13835.00	34652.50
	SD	32.22	50.60	61.11	116.99	53.90		332.81	534.46	2283.00	3929.55	6469.45
	SE	9.30	14.61	17.64	33.77	15.56		96.07	154.28	659.04	1134.36	1867.57
**Sed + Inu-R**	1	227	415	482	195	181		1410	3322.5	3397.875	0	8130
	2	221	477	748	469	311		1920	5872.5	11625	10140	29558
	3	144	390	706	561	311		1845	6060	14685	17520	40110
	4	206	350	704	596	342		1080	4815	13320	15780	34995
	5	156	389	760	762	288		1747.5	6277.5	18150	22140	48315
	6	195	393	650	549	312		1485	4897.5	12135	14130	32648
	7	156	337	646	496	244		1357.5	5032.5	12450	12840	31680
	8	172	319	650	494	274		1102.5	4687.5	12000	12720	30510
	9	142	414	694	557	300		2040	6180	14505	17190	39915
	10	197	432	656	562	336		1762.5	5205	12360	15120	34448
	11	160	386	648	490	265		1695	5355	12270	13050	32370
	12	160	532	680	471	297		2790	6690	12465	13440	35385
	MEAN	178.00	402.83	668.67	516.83	288.42		1686.25	5366.25	12446.91	13672.50	33171.91
	SD	29.83	58.93	70.51	128.86	43.89		463.79	918.56	3376.78	5283.58	9453.91
	SE	8.61	17.01	20.35	37.20	12.67		133.88	265.16	974.79	1525.24	2729.11
**Ex + Inu-R**	1	194	498	736	507	314		2280	6345	12825	12990	34440
	2	179	237	428	177	161		435	2302.5	3705.12	0	6443
	3	144	362	610	660	381		1635	5130	14730	22590	44085
	4	159	396	644	552	305		1777.5	5415	13170	16170	36533
	5	183	370	355	548	261		1402.5	2692.5	8055	13290	25440
	6	182	357	580	516	370		1312.5	4297.5	10980	15660	32250
	7	172	387	734	579	294		1612.5	5827.5	14535	15870	37845
	8	144	360	644	580	309		1620	5370	14040	18030	39060
	9	177	324	650	479	346		1102.5	4650	11625	14130	31508
	10	162	355	652	470	335		1447.5	5122.5	11970	14430	32970
	11	176	396	700	521	275		1650	5580	13035	13320	33585
	12	155	330	678	475	281		1312.5	5235	12645	13380	32573
	MEAN	168.92	364.33	617.58	505.33	302.67		1465.63	4830.63	11776.26	14155.00	32227.51
	SD	15.97	60.00	116.00	116.75	57.90		438.37	1209.78	3111.95	5216.01	9323.63
	SE	4.61	17.32	33.49	33.70	16.72		126.55	349.23	898.34	1505.73	2691.50
