# Supplementary material for: Continuous high-fat high-sugar diet overrides the therapeutic potential of fecal microbiota transplantation from exercised and/or inulin-conditioned donors in obese mice
Source: PLoS One. 2026 May 12;21(5):e0349286. doi: 10.1371/journal.pone.0349286 (PMC13166953; doi:10.1371/journal.pone.0349286)
Supplement: S1 Appendix — (ZIP) [file pone.0349286.s001.zip › Underlying data for Fig 7.pdf]

Triglyceride (mg/dL)

	Sham	Sed-R	Ex-R	Sed + Inu-R	Ex + Inu-R
1	72	61	85	79	129
2	69	75	77	67	58
3	60	40	49	76	44
4	39	38	47	59	37
5	50	73	27	43	35
6	30	27	46	32	41
7	26	38	36	29	46
8	38	51	62	105	64
9	87	64	67	69	58
10	55	37	39	37	47
11	60	43	54	55	92
12	45	47	50	32	77
Mean	52.58	49.50	53.25	56.92	60.67
SD	18.15	15.41	16.92	23.42	27.35
SE	5.24	4.45	4.88	6.76	7.90

Total cholesterol (mg/dL)

	Sham	Sed-R	Ex-R	Sed + Inu-R	Ex + Inu-R
1	216	222	235	109	231
2	183	188	228	223	214
3	199	239	200	171	218
4	167	205	242	219	195
5	138	187	161	217	215
6	185	232	214	227	230
7	164	172	228	186	179
8	196	195	157	197	181
9	196	210	214	162	182
10	183	199	240	222	223
11	181	170	250	189	194
12	159	189	210	200	221
Mean	180.58	200.67	214.92	193.50	206.92
SD	20.97	21.94	29.92	34.08	19.51
SE	6.05	6.33	8.64	9.84	5.63

FFA (μEq/L)

	Sham	Sed-R	Ex-R	Sed + Inu-R	Ex + Inu-R
1	714	795	844	638	1162
2	788	773	774	701	670
3	676	510	522	675	656
4	581	598	429	731	644
5	493	634	520	569	524
6	477	533	586	561	580
7	431	518	585	378	812
8	516	802	553	1018	646
9	666	666	692	650	502
10	590	597	669	469	552
11	508	726	668	513	707
12	578	565	593	498	517
Mean	584.83	643.08	619.58	616.75	664.33
SD	107.58	108.05	115.78	163.81	180.81
SE	31.06	31.19	33.42	47.29	52.20

AST (IU/L)

	Sham	Sed-R	Ex-R	Sed + Inu-R	Ex + Inu-R
1	55	52	60	93	58
2	42	39	36	118	59
3	50	61	46	96	68
4	54	40	73	54	43
5	35	36	45	55	108
6	50	69	53	122	85
7	35	42	55	51	37
8	58	55	112	46	49
9	48	48	57	44	46
10	41	57	66	54	57
11	45	59	55	53	40
12	79	61	52	47	59
Mean	49.33	51.58	59.17	69.42	59.08
SD	11.93	10.52	19.23	29.20	20.30
SE	3.44	3.04	5.55	8.43	5.86

ALT (IU/L)

	Sham	Sed-R	Ex-R	Sed + Inu-R	Ex + Inu-R
1	81	21	36	226	61
2	20	13	12	108	18
3	22	36	50	119	98
4	138	17	34	35	17
5	13	15	22	26	42
6	13	163	18	257	39
7	12	66	37	13	20
8	29	49	32	24	28
9	21	47	39	13	15
10	17	41	50	30	23
11	15	96	35	14	47
12	24	117	39	23	37
Mean	33.75	56.75	33.67	74.00	37.08
SD	37.75	46.61	11.53	86.17	23.81
SE	10.90	13.45	3.33	24.88	6.87
