# Supplementary material for: Continuous high-fat high-sugar diet overrides the therapeutic potential of fecal microbiota transplantation from exercised and/or inulin-conditioned donors in obese mice
Source: PLoS One. 2026 May 12;21(5):e0349286. doi: 10.1371/journal.pone.0349286 (PMC13166953; doi:10.1371/journal.pone.0349286)
Supplement: S1 Appendix — (ZIP) [file pone.0349286.s001.zip › Underlying data for Fig 8.pdf]

TNF-α	Sham	Sed-R	Ex-R	Sed + Inu-R	Ex + Inu-R
1	0.48	0.60	0.63	0.74	0.52
2	1.45	1.08	0.67	0.45	0.76
3	1.37	0.97	0.82	0.55	1.20
4	0.77	0.62	0.59	1.16	0.55
5	1.04	1.02	0.67	1.15	0.64
6	0.89	0.53	1.01	1.11	0.76
7	1.13	0.70	0.81	0.81	0.64
8	0.87	0.72	0.70	0.95	2.49
MEAN	1.00	0.78	0.74	0.86	0.94
SD	0.32	0.21	0.14	0.28	0.66
SE	0.11	0.07	0.05	0.10	0.23

IL-1β	Sham	Sed-R	Ex-R	Sed + Inu-R	Ex + Inu-R
1	0.57	0.73	0.34	0.65	0.60
2	0.80	0.61	0.60	0.72	0.84
3	1.04	0.82	0.81	0.46	0.77
4	0.76	0.96	0.88	0.94	0.94
5	1.75	0.80	0.50	1.01	0.56
6	1.05	0.91	1.24	0.69	0.79
7	1.05	0.99	0.82	1.98	0.73
8	0.97	1.17	0.91	0.77	0.78
MEAN	1.00	0.87	0.76	0.90	0.75
SD	0.35	0.17	0.28	0.47	0.12
SE	0.12	0.06	0.10	0.16	0.04
