# Supplementary material for: Continuous high-fat high-sugar diet overrides the therapeutic potential of fecal microbiota transplantation from exercised and/or inulin-conditioned donors in obese mice
Source: PLoS One. 2026 May 12;21(5):e0349286. doi: 10.1371/journal.pone.0349286 (PMC13166953; doi:10.1371/journal.pone.0349286)
Supplement: S1 Appendix — (ZIP) [file pone.0349286.s001.zip › Underlying data for Fig 9.pdf]

"Donor" SCFAs

Acetate (mg/cecum)

	Sed	Ex	Sed + Inu	Ex + Inu
1	0.42	0.46	1.26	0.63
2	0.52	0.53	1.12	0.97
3	0.28	0.68	1.53	1.05
4	0.54	0.18	1.46	1.35
5	0.47	0.51	1.57	1.28
6	0.66	0.34	1.15	0.83
Mean	0.48	0.45	1.35	1.02
SD	0.13	0.17	0.20	0.27
SE	0.05	0.07	0.08	0.11

Propionate (mg/cecum)

	Sed	Ex	Sed + Inu	Ex + Inu
1	0.21	0.15	0.29	0.30
2	0.36	0.26	0.59	0.29
3	0.17	0.22	0.32	0.43
4	0.24	0.13	0.33	0.27
5	0.35	0.42	0.62	0.32
6	0.37	0.16	0.35	0.31
Mean	0.28	0.22	0.42	0.32
SD	0.09	0.11	0.15	0.06
SE	0.03	0.04	0.06	0.02

Butyrate (mg/cecum)

	Sed	Ex	Sed + Inu	Ex + Inu
1	0.18	0.20	0.58	0.53
2	0.40	0.28	0.76	0.79
3	0.12	0.23	0.49	0.74
4	0.30	0.11	0.51	1.02
5	0.34	0.48	1.13	1.13
6	0.46	0.09	0.58	0.52
Mean	0.30	0.23	0.67	0.79
SD	0.13	0.14	0.24	0.25
SE	0.05	0.06	0.10	0.10

"Recipient" SCFAs

Acetate (mg/cecum)

	Sham	Sed-R	Ex-R	Sed + Inu-R	Ex + Inu-R
1	0.28	0.39	0.43	0.27	0.28
2	0.28	0.21	0.48	0.27	0.47
3	0.24	0.22	0.42	0.36	0.29
4	0.17	0.35	0.28	0.29	0.33
5	0.24	0.19	0.27	0.34	0.23
6	0.36	0.44	0.60	0.38	0.16
7	0.28	0.29	0.35	0.42	0.52
8	0.35	0.27	0.33	0.22	0.30
Mean	0.27	0.30	0.40	0.32	0.32
SD	0.06	0.09	0.11	0.07	0.12
SE	0.02	0.03	0.04	0.02	0.04

Propionate (mg/cecum)

	Sham	Sed-R	Ex-R	Sed + Inu-R	Ex + Inu-R
1	0.11	0.11	0.12	0.11	0.13
2	0.10	0.12	0.17	0.15	0.25
3	0.09	0.08	0.19	0.15	0.13
4	0.05	0.09	0.11	0.12	0.14
5	0.03	0.07	0.12	0.18	0.11
6	0.03	0.09	0.27	0.14	0.10
7	0.08	0.10	0.12	0.15	0.14
8	0.11	0.10	0.12	0.09	0.11
Mean	0.08	0.10	0.15	0.14	0.14
SD	0.04	0.02	0.06	0.03	0.05
SE	0.01	0.01	0.02	0.01	0.02

Butyrate (mg/cecum)

	Sham	Sed-R	Ex-R	Sed + Inu-R	Ex + Inu-R
1	0.09	0.14	0.11	0.11	0.05
2	0.12	0.10	0.11	0.10	0.09
3	0.07	0.09	0.10	0.11	0.06
4	0.08	0.10	0.10	0.09	0.06
5	0.13	0.09	0.09	0.08	0.08
6	0.11	0.12	0.11	0.06	0.05
7	0.09	0.09	0.09	0.08	0.08
8	0.10	0.12	0.09	0.07	0.07
Mean	0.10	0.10	0.10	0.09	0.07
SD	0.02	0.02	0.01	0.02	0.01
SE	0.01	0.01	0.00	0.01	0.00
